# Comparison of estimated and measured GFR in pediatric CKD patients transitioning from adolescence to adulthood: results from KNOW-PedCKD

**DOI:** 10.1186/s12882-026-04942-w

**Published:** 2026-04-02

**Authors:** Seon Hee Lim, Eujin Park, Kyung Hee Han, Ji Yeon Song, Seong Heon Kim, Naye Choi, Heeyeon Cho, Jeong Yeon Kim, Jae Il Shin, Keum Hwa Lee, Min Hyun Cho, Min Ji Park, Hee Sun Baek, Joo Hoon Lee, Jiwon Jung, Eun Mi Yang, Ji Hyun Kim, Il-Soo Ha, Hee Gyung Kang, Yo Han Ahn

**Affiliations:** 1 Department of Pediatrics, Pusan National University Children’s Hospital, Yangsan, Republic of Korea; 2 Department of Pediatrics, Korea University Guro Hospital, Seoul, Republic of Korea; 3 Department of Pediatrics, Jeju University Hospital, Jeju, Republic of Korea; 4 Department of Pediatrics, Seoul National University Children’s Hospital, Seoul, Republic of Korea; 5 Kidney Research Institute, Seoul National University Medical Research Center, Seoul, Republic of Korea; 6 Department of Pediatrics, Samsung Medical Center, Sungkyunkwan University School of Medicine, Seoul, Republic of Korea; 7 Division of Pediatric Nephrology, Severance Children’s Hospital, Seoul, Republic of Korea; 8 Department of Pediatrics, School of Medicine, Kyungpook National University, Daegu, Republic of Korea; 9 Department of Pediatrics, Asan Medical Center Children’s Hospital, Ulsan University, College of Medicine, Seoul, Republic of Korea; 10 Department of Pediatrics, Chonnam National University Hospital and Medical School, Gwangju, South Korea; 11 Department of Pediatrics, Seoul National University Bundang Hospital, Seongnam, South Korea; 12 Department of Pediatrics, Seoul National University College of Medicine, Seoul, Republic of Korea; 13 Wide River Institute of Immunology, Seoul National University, Hongcheon, Republic of Korea

**Keywords:** Chronic kidney disease, Glomerular filtration rate, Adolescence

## Abstract

**Background:**

Accurate estimation of glomerular filtration rate (eGFR) is essential for managing pediatric chronic kidney disease (CKD). While multiple eGFR equations are used clinically, their reliability in adolescents transitioning to adulthood with pediatric-onset CKD remains uncertain. This study aimed to evaluate the accuracy of 10 eGFR equations against measured GFR (mGFR) values in South Korean adolescents and young adults with CKD using data from the KoreaN Cohort Study for Outcomes in Patients With Pediatric Chronic Kidney Disease (KNOW-PedCKD) cohort.

**Methods:**

Patients aged ≥ 15 years who underwent mGFR testing were included in the KNOW-PedCKD study. mGFR was determined using plasma clearance of ^51^Cr-EDTA or ^99m^Tc-DTPA. Ten eGFR equations (U25_Cr_, U25_CysC_, U25_Cr-CysC_, Schwartz_Cr_, CKiD_Cr-CysC_, FAS_Cr_-Age, FAS_Cr_-Ht, FAS_CysC_, CKD-EPI_Cr_, and CKD-EPI_Cr-CysC_) were compared with concurrent mGFR values. Performance was assessed using bias, precision, and accuracy expressed as the percentage of eGFR estimates within 10% [P10] and 30% [P30] of the mGFR.

**Results:**

The analysis encompassed 187 mGFR measurements from 82 patients (median age 18.4 years, interquartile range (IQR) 16.5–20.8; 75.9% male). Median mGFR was 42.3 (19.3–70.5) mL/min/1.73 m^2^. Overall, the U25_Cr-CysC_ showed the most balanced performance, with low bias (1.9 mL/min/1.73 m^2^), high precision (SD 10.5), and accuracy values of 32.6% for P10 and 67.4% for P30. FAS_Cr_-Ht equation exhibited the highest accuracy (77.0%) and the lowest bias (-0.44), with slightly lower precision (SD 12.5). Both equations performed constantly across adolescents and young adult subgroups. In contrast, CKD-EPI_Cr_ equation consistently overestimated GFR with the highest bias and lowest accuracy. None of the evaluated equations achieved accuracy of 80–90% within 30% of mGFR, which are generally considered acceptable. However, after exclusion of measurements corresponding to CKD stage 5, the FAS_Cr_-Ht and Schwartz_Cr_ equations achieved P30 accuracies of 84.6% and 84.0%, respectively.

**Conclusions:**

The eGFR equations covering children — particularly U25_Cr-CysC_ and FAS_Cr_-Ht equations — provide more reliably estimates of GFR than adult equations in Korean adolescents and young adults with CKD. These findings support the use of age-spanning formulas during transitional care to improve clinical accuracy and continuity.

**Supplementary Information:**

The online version contains supplementary material available at 10.1186/s12882-026-04942-w.

## Background

Glomerular filtration rate (GFR) is a standard measure of kidney function. Although inulin clearance is considered the gold standard, it is rarely used in clinical practice owing to its complexity. The measured GFR (mGFR) using isotope tracers serves as a reliable reference method; however, estimated GFR (eGFR) equations are more practical and widely adopted in routine care.

Accurate estimation of GFR is essential for managing pediatric chronic kidney disease (CKD) and guiding diagnosis, staging, treatment decisions, and prognostic assessments, all of which significantly influence long-term outcomes and quality of life [1, 2]. Among the various pediatric eGFR equations, the Schwartz equation, particularly the 2012 version, is the most validated and commonly used for children and adolescents with mild to moderate CKD [3]. In adults, the 2021 CKD Epidemiology Collaboration (CKD-EPI) equation is widely used; it estimates GFR based on creatinine, age, and sex and does not include race as a variable [4].

However, there is no consensus regarding the optimal eGFR equation for individuals transitioning from adolescence to early adulthood. Studies have shown persistent discrepancies between pediatric and adult equations extending into the third decades of life [5, 6]. Such discontinuities can complicate clinical decision making and lead to confusion during the transition from pediatric to adult care. To address this gap, age-spanning equations such as the Full Age Spectrum (FAS) and the U25 equations developed by the Chronic Kidney Disease in Children (CKiD) Study have been proposed [7, 8].

This study aimed to compare ten different eGFR equations with corresponding mGFR determinations in Korean pediatric CKD patients aged 15 years and older using data from the KoreaN cohort study for Outcomes in patients With Pediatric CKD (KNOW-PedCKD) cohort to identify the equation that most accurately reflects true kidney function during the transition from adolescence to early adulthood.

## Materials and methods

### Study participants and definitions

CKD patients aged ≥ 15 years who underwent mGFR testing were enrolled in this study from the KNOW-PedCKD, a multicenter, prospective, observational cohort study (ClinicalTrials.gov: NCT02165878; date of registration: June 11, 2014) [9, 10]. Recruitment was conducted between 2011 and 2016 at seven major pediatric nephrology centers in South Korea. Enrolled participants underwent annual assessments, including anthropometric measurements, laboratory tests, kidney ultrasonography, cardiovascular assessments, metabolic bone disease marker detection, and standardized questionnaires, as previously described [9, 10]. Isotope GFR measurements were conducted triennially during the follow-up period.

The etiology of CKD was classified as glomerulopathy or nonglomerulopathy. CKD was defined and staged using mGFR values in accordance with the Kidney Disease Improving Global Outcomes (KDIGO) guidelines [11]. Height, weight, and body mass index (BMI) Z-scores were calculated using the 2017 Korean National Pediatric and Adolescent Standard Growth Charts [12].

### mGFR and eGFR

The mGFR was determined using plasma clearance of either chromium-51 ethylenediaminetetraacetic acid (^51^Cr-EDTA) or technetium-99m diethylenetriamine pentaacetic acid (^99m^Tc-DTPA) depending on availability at each participating center. Following the discontinuation of ^51^Cr-EDTA production at the end of 2018, only ^99m^Tc-DTPA was available across all participating centers from March 2019 onward. Both methods are well-established reference standards for GFR measurement and have demonstrated comparable accuracy in previous validation studies [13–16]. Tracer-specific standardized plasma sampling protocols were applied across participating centers. Blood samples were collected at 3 and 5 h after ^51^Cr-EDTA injection and at 2 and 4 h after ^99m^Tc-DTPA injection. The gamma activity of each tracer in plasma samples and standard solutions was measured using a gamma counter with tracer-specific calibration settings. Tracer measurements were performed at each participating center according to institutional nuclear medicine protocols and routine quality control procedures. Plasma clearance was calculated using the slope-intercept method based on a one-compartment pharmacokinetic model. To account for the late distribution phase and slow equilibrating compartment, clearance values were corrected using the Brøchner-Mortensen and Rødbro correction, which provides a validated approximation of two-compartment kinetics [17–19]. The mGFR was expressed as plasma clearance normalized to body surface area, which was calculated using the DuBois method based on height and weight measurements obtained at the time of mGFR assessment [20].

At the time of mGFR measurement, serum blood urea nitrogen (BUN), creatinine (Cr), and cystatin C (CysC) levels were measured in a central laboratory. Serum BUN levels were determined using the urease/glutamate dehydrogenase method (Siemens ADVIA Chemistry; Siemens Healthcare Diagnostics Inc., Tarrytown, NY, USA). Serum creatinine was measured using the Jaffe rate blank method with IDMS-traceable calibration (ADIVA Chemistry Creatinine 2, Siemens, Germany). Cystatin C was measured using a latex-enhanced nephelometry method (BN II System N Latex Cystatin C, Siemens, Germany) calibrated to the international reference material (ERM^®^-DA471/IFCC). The central laboratory maintained internal quality control procedures and participated in external proficiency testing programs to ensure long-term analytical accuracy and traceability. Because all samples from participating centers were analyzed using a single analytical platform and calibration system at the central laboratory, inter-laboratory variability and lot-to-lot calibration differences were minimized.

A total of 10 eGFR equations were evaluated as follows: Cr-based U25 (U25_Cr_), CysC-based U25 (U25_CysC_), Cr-CysC-based U25 (U25_Cr−CysC_), Schwartz_Cr_, CKiD_Cr−CysC_, FAS Age Cr (FAS_Cr_-Age), FAS Height Cr (FAS_Cr_-Ht), FAS CysC (FAS_CysC_), Cr-based CKD-EPI (CKD-EPI_Cr_), and Cr-CysC based CKD-EPI (CKD-EPI_Cr−CysC_) equations [3, 8, 21–23]. The formulae for these equations are provided in the Supplementary Material. The cystatin C-based and creatinine-cystatin C-based eGFR equations included in this study were derived using IFCC-standardized cystatin C assays. Therefore, no additional recalibration of cystatin C was required for equation application in this cohort.

### Statistical analysis

Statistical analyses were performed using SPSS software v.25 (IBM Corp., Armonk, NY, USA). Performance of the eGFR equations was assessed in comparison with that of the mGFR in terms of bias, precision, and accuracy. Bias was defined as the mean difference between mGFR and eGFR. Precision was defined as the standard deviation (SD) of the difference. Accuracy was assessed as the percentage of eGFR values within 10% (P10) and 30% (P30) of mGFR.

Continuous variables were summarized as mean ± SD or median with interquartile range (IQR), as appropriate. Categorical variables were compared using the chi-square test. Spearman’s correlation coefficients were calculated to assess the relationship between mGFR and eGFR. Agreement between mGFR and eGFR was further evaluated using Bland-Altman plots. Limits of agreement were calculated as the mean difference (bias) ± 1.96 × SD of the differences. Proportional bias was assessed by linear regression analysis of the difference between mGFR and eGFR against their mean values. A regression slope significantly different from zero was considered evidence of proportional bias. To assess heteroscedasticity, linear regression analysis of absolute error against mean GFR was performed. *P* < 0.05 was considered statistically significant.

## Results

### Baseline characteristics

The number of enrolled patients was 82, 74.4% of whom were men. The majority (80.5%) of patients had non-glomerular causes of CKD. Each participant underwent a median of two mGFR assessments (IQR, 1–3), resulting in 187 person-visits being included in the analysis (Table 1). The median age was 18.4 years old (IQR 16.5–20.8), and 75.9% of visits were from male patients (*n* = 142). Median height and weight were 164.4 cm (IQR, 158.0–171.6) and 59.8 kg (IQR, 49.3–68.3), respectively. The median mGFR was 42.3 mL/min/1.73 m^2^ (IQR, 19.3–70.5), with CKD stage distribution as follows: stage 1, 13.9%; stage 2, 19.3%; stage 3, 28.9%; stage 4, 21.4%; and stage 5, 16.6%.

**Table 1:** Baseline characteristics of 187 person-visits

Characteristics	Values
Sex, male: female	142:45
Age, years	18.4 (16.5–20.8)
Height, cm	164.4 (158.0–171.6)
Body weight, kg	59.8 (49.3–68.3)
Body mass index, kg/m^2^	21.6 (18.9–25.4)
Height Z score	-1.0 (-1.7– -0.1)
Weight Z score	-0.3 (-1.3–0.6)
Body mass index Z score	-0.02 (-0.9–1.2)
Underlying disease	
Non-glomerular	148 (79.1)
Glomerular	39 (20.9)
BUN, mg/dL	26.7 (19.0–42.7)
Creatinine, mg/dL	1.6 (1.2–3.2)
Cystatin C, mg/L	1.6 (1.2–2.7)
Measured GFR, mL/min/1.73 m^2^	42.3 (19.3–70.5)
Chronic kidney disease stage by measured GFR	
Stage 1	26 (13.9)
Stage 2	36 (19.3)
Stage 3	54 (28.9)
Stage 4	40 (21.4)
Stage 5	31 (16.6)

Variables are presented as median (interquartile range) or numbers (%)

### Overall performance of eGFR equations

Among the 10 evaluated eGFR equations, U25_Cr−CysC_ demonstrated the most balanced performance, with low bias (1.9 mL/min/1.73 m^2^), high precision (SD 10.5), P10 of 32.6% and P30 of 67.4%, and a strong correlation coefficient (ρ = 0.97) (Table 2). FAS_Cr_-Ht achieved the highest P30 (77.0%) and the lowest bias (-0.44), albeit with slightly lower precision. In contrast, CKD-EPI_Cr_ demonstrated the greatest overestimation, with the highest bias (14.7) and lowest P10 (14.4%) and P30 (43.9%), respectively. Similarly, FAS_Cr_-Age and FAS_CysC_ exhibited relatively high biases (8.3 and 9.7, respectively) and lower P30 values (52.9% and 49.2%, respectively). All equations demonstrated strong correlations with mGFR (ρ ≥ 0.93, *P* < 0.001) (Supplementary Fig. 1, Supplementary Table 1).

**Table 2:** Prediction performance results of different eGFR on measured GFR

	mL/min/1.73m^2^	Bias	Precision	P10, *n* (%)	P30, *n* (%)
U25_Cr_	50.9 ± 29.0	2.6	10.9	52 (27.8)	133 (71.1)
U25_CysC_	49.5 ± 25.5	1.2	12.8	56 (29.9)	117 (62.6)
U25_Cr−CysC_	50.2 ± 26.8	1.9	10.5	61 (32.6)	126 (67.4)
Schwartz_Cr_	44.9 ± 26.7	-3.4	12.6	51 (27.3)	143 (76.5)
CKiD_Cr−CysC_	51.6 ± 26.2	3.3	10.6	57 (30.5)	112 (59.9)
FAS_Cr_-Age	56.6 ± 32.9	8.3	12.8	38 (20.3)	99 (52.9)
FAS_Cr_-Ht	47.9 ± 28.7	-0.44	12.5	63 (33.7)	144 (77.0)
FAS_CysC_	58.1 ± 29.9	9.7	12.8	45 (24.1)	92 (49.2)
CKD-EPI_Cr_	63.0 ± 38.7	14.7	13.7	27 (14.4)	82 (43.9)
CKD-EPI_Cr−CysC_	56.3 ± 35.6	8.0	9.5	49 (26.2)	122 (65.2)

P10, percentage of eGFR values within 10% of mGFR; P30, percentage of eGFR values within 30% of mGFR; Cr, creatinine; CysC, cystatin C; CKiD, Chronic Kidney Disease in Chidren; FAS, Full Age Spectrum; Ht, height; CKD-EPI, Chronic Kidney Disease Epidemiology Collaboration

### Sex- and age-specific analysis

The performance of the eGFR equations was generally consistent across sexes, with some differences observed (Table 3). U25_Cr−CysC_ exhibited a similar bias and precision in males (bias 1.9; SD 9.9) and females (bias 1.7; SD 12.4), although P30 was higher in males (69.7%) than in females (60%). Most equations showed slightly higher correlation coefficients in females, with CKiD_Cr−CysC_ achieving the highest in females (ρ = 0.99, Supplementary Table 1).

**Table 3:** Bias, precision and accuracy of eGFR on measured GFR by sex

	mL/min/1.73m^2^		Bias		Precision		P10, *n* (%)		P30, *n* (%)	
	male (*n* = 142)	female (*n* = 45)	male (*n* = 142)	female (*n* = 45)	male (*n* = 142)	female (*n* = 45)	male (*n* = 142)	female (*n* = 45)	male (*n* = 142)	female (*n* = 45)
U25_Cr_	51.3 ± 26.8	49.4 ± 35.4	2.4	3.1	11.1	10.6	43 (30.3)	9 (20)	103 (72.5)	30 (66.7)
U25_CysC_	50.4 ± 23.8	46.7 ± 30.4	1.4	0.41	11.0	17.5	47 (33.1)	9 (20)	90 (63.4)	27 (60)
U25_Cr−CysC_	50.8 ± 24.9	48.1 ± 32.2	1.9	1.7	9.9	12.4	47 (33.1)	14 (31.1)	99 (69.7)	27 (60)
Schwartz_Cr_	43.2 ± 23.0	50.0 ± 35.9	-5.7	3.7	12.3	10.9	41 (28.9)	10 (22.2)	112 (78.9)	31 (68.9)
CKiD_Cr−CysC_	51.6 ± 23.9	51.6 ± 32.6	2.7	5.3	10.3	11.5	44 (31.0)	13 (28.9)	92 (64.8)	20 (44.4)
FAS_Cr_-Age	56.0 ± 29.7	58.5 ± 41.7	7.0	12.2	13.5	9.3	32 (22.5)	6 (13.3)	84 (59.2)	15 (33.3)
FAS_Cr_-Ht	47.3 ± 26.1	49.6 ± 36.0	-1.6	3.3	12.7	10.9	52 (36.6)	11 (24.4)	113 (79.6)	31 (68.9)
FAS_CysC_	57.2 ± 26.1	60.7 ± 39.7	8.3	14.4	10.3	17.3	36 (25.4)	9 (20)	75 (52.8)	17 (37.8)
CKD-EPI_Cr_	64.6 ± 37.9	58.2 ± 41.3	15.6	11.8	14.2	11.8	24 (16.9)	3 (6.7)	65 (45.8)	17 (37.8)
CKD-EPI_Cr−CysC_	57.1 ± 33.8	53.8 ± 41.1	8.2	7.5	9.5	9.6	37 (26.1)	12 (26.7)	96 (67.6)	26 (57.8)

P10, percentage of eGFR values within 10% of mGFR; P30, percentage of eGFR values within 30% of mGFR; Cr, creatinine; CysC, cystatin C; CKiD, Chronic Kidney Disease in Chidren; FAS, Full Age Spectrum; Ht, height; CKD-EPI, Chronic Kidney Disease Epidemiology Collaboration

Age group analysis revealed no substantial differences in performance between adolescents (15–18 years) and young adults (≥ 18 years) (Table 4). U25_Cr−CysC_ maintained consistent performance across age groups, with a P30 of 67% in both subgroups. FAS_Cr_-Ht also performed well in both age groups, with P30 values of 77.1% in adolescents and 76.9% in young adults. CKD-EPI_Cr_ consistently overestimated the mGFR and exhibited the largest bias and lowest accuracy in both age groups.

**Table 4:** Bias, precision and accuracy of eGFR on measured GFR by age

	mL/min/1.73m^2^		Bias		Precision		P10, *n* (%)		P30, *n* (%)	
	15–18 yrs (*n* = 83)	≥ 18yrs (*n* = 104)	15–18 yrs (*n* = 83)	≥ 18yrs (*n* = 104)	15–18 yrs (*n* = 83)	≥ 18yrs (*n* = 104)	15–18 yrs (*n* = 83)	≥ 18yrs (*n* = 104)	15–18 yrs (*n* = 83)	≥ 18yrs (*n* = 104)
U25_Cr_	50.6 ± 30.8	51.1 ± 27.8	2.0	3.0	11.7	10.3	19 (22.9)	33 (31.7)	62 (74.7)	71 (68.3)
U25_CysC_	50.0 ± 27.4	49.1 ± 24.1	1.3	1.1	13.0	12.7	27 (32.5)	29 (27.9)	52 (62.7)	65 (62.5)
U25_Cr−CysC_	50.3 ± 28.4	50.1 ± 25.5	1.7	2.0	10.6	10.5	26 (31.3)	35 (33.7)	56 (67.5)	70 (67.3)
Schwartz_Cr_	46.0 ± 28.6	43.9 ± 25.3	-2.6	-4.1	13.2	12.1	26 (31.3)	25 (24.0)	64 (77.1)	79 (76.0)
CKiD_Cr−CysC_	51.9 ± 27.5	51.4 ± 25.2	3.3	3.3	10.9	10.4	25 (30.1)	32 (30.8)	48 (57.8)	64 (61.5)
FAS_Cr_-Age	55.1 ± 33.4	57.8 ± 32.6	6.4	9.8	12.7	12.7	17 (20.5)	21 (20.2)	51 (61.4)	48 (46.2)
FAS_Cr_-Ht	49.5 ± 31.2	46.6 ± 26.5	0.87	-1.5	12.7	12.3	29 (34.9)	34 (32.7)	64 (77.1)	80 (76.9)
FAS_CysC_	57.4 ± 31.3	58.6 ± 28.8	8.7	10.6	15.7	9.8	24 (28.9)	21 (20.2)	44 (53.0)	48 (46.2)
CKD-EPI_Cr_	65.5 ± 42.4	61.0 ± 35.6	16.9	13.0	14.4	13.0	10 (12.0)	17 (16.3)	36 (43.4)	46 (44.2)
CKD-EPI_Cr−CysC_	57.3 ± 38.1	55.6 ± 33.6	8.7	7.5	9.9	9.2	22 (26.5)	27 (26.0)	52 (62.7)	70 (67.3)

P10, percentage of eGFR values within 10% of mGFR; P30, percentage of eGFR values within 30% of mGFR; yrs, years; Cr, creatinine; CysC, cystatin C; CKiD, Chronic Kidney Disease in Chidren; FAS, Full Age Spectrum; Ht, height; CKD-EPI, Chronic Kidney Disease Epidemiology Collaboration

## Performance by CKD stage

The performance of the eGFR equations varied across CKD stages (Table 5). In stages 1 and 2, most equations, particularly the Schwartz_Cr_, CKiD_Cr−CysC_, FAS_Cr_-Ht, and U25 equations, tended to underestimate mGFR. Conversely, in stages 3, 4, and 5, most equations overestimated the mGFR. The CKD-EPI equations overestimated GFR across all CKD stages.

**Table 5:** Bias, precision and accuracy of eGFR on measured GFR by CKD stage

	mL/min/1.73m^2^	Bias	Precision	P10, *n* (%)	P30, *n* (%)
CKD stage 1 (*n* = 26)					
U25_Cr_	99.1 ± 18.5	-9.2	16.7	6 (23.1)	25 (96.2)
U25_CysC_	91.6 ± 14.0	-16.6	12.2	9 (34.6)	26 (100)
U25_Cr−CysC_	95.4 ± 15.0	-12.9	13.0	10 (38.5)	26 (100)
Schwartz_Cr_	90.9 ± 22.6	-17.3	17.9	8 (30.8)	21 (80.8)
CKiD_Cr−CysC_	95.7 ± 15.6	-12.6	11.2	12 (46.2)	26 (100)
FAS_Cr_-Age	110.4 ± 24.5	2.2	18.6	9 (34.6)	24 (92.3)
FAS_Cr_-Ht	96.0 ± 21.4	-12.2	18.6	10 (38.5)	23 (88.5)
FAS_CysC_	106.0 ± 19.9	-2.2	9.9	19 (73.1)	26 (100)
CKD-EPI_Cr_	126.0 ± 13.9	17.8	18.4	8 (30.8)	19 (73.1)
CKD-EPI_Cr−CysC_	117.8 ± 15.2	9.6	12.6	14 (53.8)	25 (96.2)
CKD stage 2 (*n* = 36)					
U25_Cr_	70.9 ± 11.3	-2.4	9.1	23 (63.9)	35 (97.2)
U25_CysC_	67.9 ± 10.2	-5.5	10.6	18 (50)	34 (94.4)
U25_Cr−CysC_	69.4 ± 8.4	-3.9	7.2	21 (58.3)	36 (100)
Schwartz_Cr_	60.1 ± 10.0	-13.3	9.4	3 (8.3)	32 (88.9)
CKiD_Cr−CysC_	70.1 ± 7.8	-3.3	7.8	23 (63.9)	36 (100)
FAS_Cr_-Age	75.7 ± 17.7	2.3	15.2	17 (47.2)	32 (88.9)
FAS_Cr_-Ht	66.6 ± 13.1	-6.7	12.8	12 (33.3)	34 (94.4)
FAS_CysC_	77.5 ± 11.5	4.2	12.0	16 (44.4)	32 (88.9)
CKD-EPI_Cr_	91.1 ± 17.2	17.8	13.6	5 (13.9)	22 (61.1)
CKD-EPI_Cr−CysC_	81.8 ± 12.7	8.5	10.6	12 (33.3)	31 (86.1)
CKD stage 3 (*n* = 54)					
U25_Cr_	51.5 ± 10.7	6.4	8.2	15 (27.8)	44 (81.5)
U25_CysC_	48.7 ± 13.4	3.5	11.3	26 (48.1)	45 (83.3)
U25_Cr−CysC_	50.1 ± 10.4	5.0	7.7	23 (42.6)	46 (85.2)
Schwartz_Cr_	45.3 ± 8.7	0.11	7.7	25 (46.3)	51 (94.4)
CKiD_Cr−CysC_	51.9 ± 9.7	6.7	6.7	19 (35.2)	42 (77.8)
FAS_Cr_-Age	59.3 ± 13.0	14.1	11.1	8 (14.8)	25 (46.3)
FAS_Cr_-Ht	47.1 ± 10.5	2.0	8.6	28 (51.9)	48 (88.9)
FAS_CysC_	58.5 ± 18.7	13.4	16.4	9 (16.7)	30 (55.6)
CKD-EPI_Cr_	64.8 ± 16.8	19.7	13.7	2 (3.7)	18 (33.3)
CKD-EPI_Cr−CysC_	55.4 ± 14.7	10.2	10.8	13 (24.1)	39 (72.2)
CKD stage 4 (*n* = 40)					
U25_Cr_	28.1 ± 8.1	7.2	7.2	8 (20)	20 (50)
U25_CysC_	29.4 ± 6.0	8.6	4.7	3 (7.5)	12 (30)
U25_Cr−CysC_	28.7 ± 6.5	7.9	5.3	6 (15)	15 (37.5)
Schwartz_Cr_	24.7 ± 8.1	3.9	7.1	9 (22.5)	27 (67.5)
CKiD_Cr−CysC_	30.9 ± 6.8	10.1	5.5	3 (7.5)	7 (17.5)
FAS_Cr_-Age	31.0 ± 8.8	10.2	7.7	4 (10)	14 (35)
FAS_Cr_-Ht	26.6 ± 9.0	5.8	8.2	9 (22.5)	27 (67.5)
FAS_CysC_	34.7 ± 7.0	13.8	5.5	1 (2.5)	4 (10)
CKD-EPI_Cr_	31.2 ± 12.3	10.4	10.8	8 (20)	15 (37.5)
CKD-EPI_Cr−CysC_	26.9 ± 8.1	6.1	6.4	6 (15)	18 (45)
CKD stage 5 (*n* = 31)					
U25_Cr_	15.4 ± 4.6	5.5	3.6	0 (0)	9 (29.0)
U25_CysC_	20.1 ± 4.1	10.2	3.0	0 (0)	0 (0)
U25_Cr−CysC_	17.8 ± 4.1	7.8	3.1	1 (3.2)	3 (9.7)
Schwartz_Cr_	13.9 ± 4.3	3.9	3.7	6 (19.4)	12 (38.7)
CKiD_Cr−CysC_	19.5 ± 4.4	9.6	3.4	0 (0)	1 (3.2)
FAS_Cr_-Age	17.5 ± 4.9	7.6	3.9	0 (0)	4 (12.9)
FAS_Cr_-Ht	14.4 ± 4.6	4.5	3.9	4 (12.9)	12 (38.7)
FAS_CysC_	24.6 ± 4.7	14.6	3.9	0 (0)	0 (0)
CKD-EPI_Cr_	15.5 ± 5.2	5.6	4.1	4 (12.9)	8 (25.8)
CKD-EPI_Cr−CysC_	14.9 ± 4.3	5.0	3.4	4 (12.9)	9 (29.0)

P10, percentage of eGFR values within 10% of mGFR; P30, percentage of eGFR values within 30% of mGFR; CKD, chronic kidney disease; Cr, creatinine; CysC, cystatin C; CKiD, Chronic Kidney Disease in Chidren; FAS, Full Age Spectrum; Ht, height; CKD-EPI, Chronic Kidney Disease Epidemiology Collaboration

The most appropriate equations for each CKD stage based on the combined assessment of bias, precision, and accuracy were as follows: FAS_CysC_ in stage 1; U25_Cr_, U25_Cr−CysC_, and CKiD_Cr−CysC_ in stage 2; and Schwartz_Cr_ and FAS_Cr_-Ht in stage 3. In Stage 4, the performance declined across all equations. In stage 5 cases, no equation demonstrated acceptable agreement with mGFR. When CKD stage 5 visits were excluded, FAS_Cr_-Ht and Schwartz_Cr_ equations achieved P30 accuracies of 84.6% and 84.0%, respectively.

### Bland-Altman and correlation analyses

**Fig. 1:**
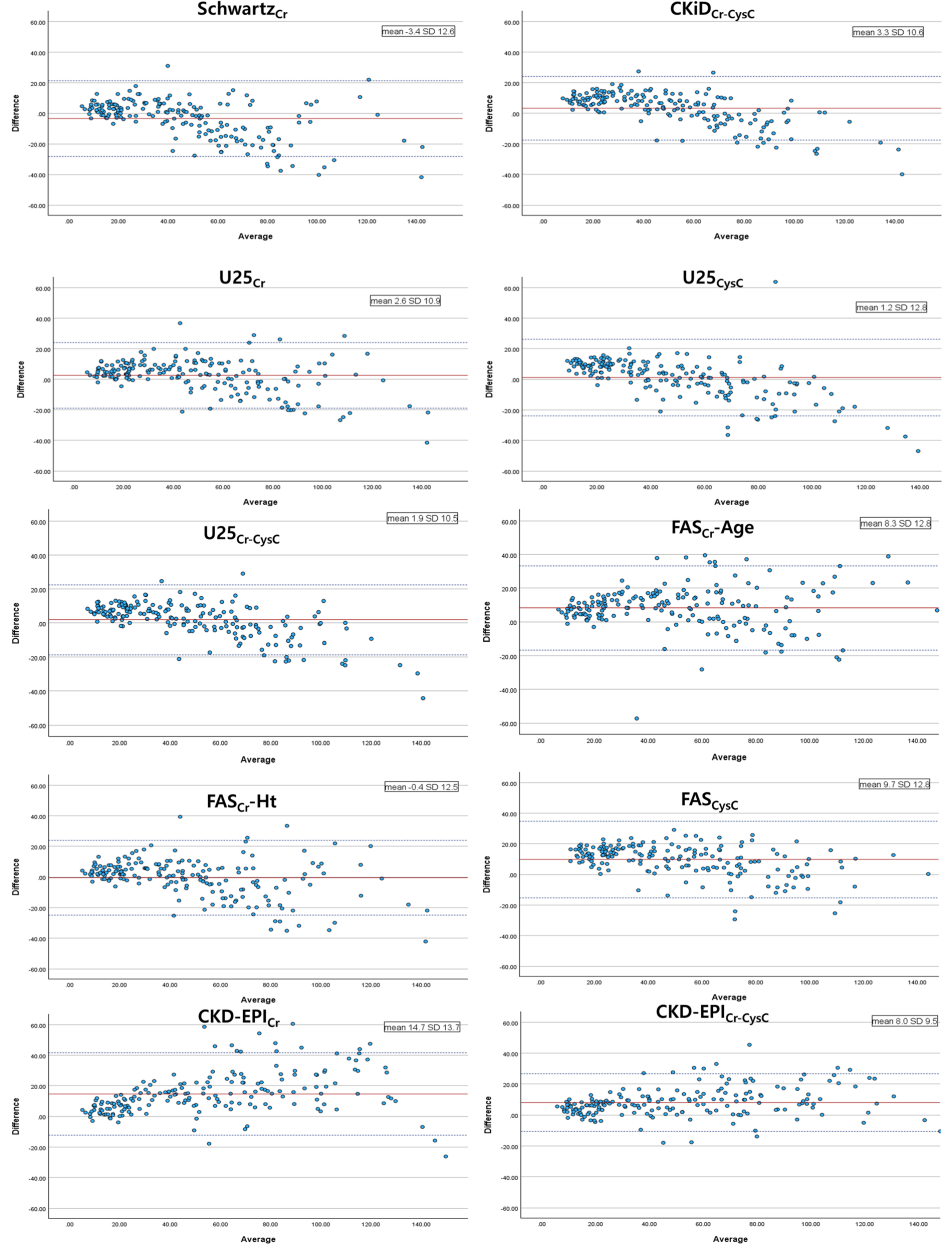
Bland-Altman plots showing the disagreement between eGFR and mGFR

Bland–Altman plots (Fig. 1) showed increasing disagreement between eGFR and mGFR at higher GFR levels. Significant proportional bias was observed in all equations except the FAS_Cr_-Age equation, as indicated by regression slopes significantly different from zero (Supplementary Table 2). Most pediatric equations demonstrated negative slopes, indicating increasing underestimation of GFR at higher mGFR values. In contrast, the CKD-EPI_Cr_ and CKD-EPI_Cr−CysC_ equations exhibited positive slopes, suggesting increasing overestimation at higher GFR levels. Regression analysis of absolute error demonstrated that estimation variability increased significantly with higher mGFR levels in all eGFR equations except FAS_CysC_, indicating increasing heteroscedasticity (Supplementary Table 3).

When stratified by biomarker type, creatinine-only equations showed wider limits of agreement, particularly for CKD-EPI_Cr_, suggesting greater variability in estimation error. Combined creatinine-cystatin C equations demonstrated more consistent agreement with mGFR and smaller increases in absolute error compared with most single-marker equations (Supplementary Table 2).

Spearman correlation analysis (Supplementary Fig. 1) confirmed strong correlations between mGFR and most eGFR estimates.

## Discussions

This study evaluated the performance of various eGFR equations against mGFR in patients with CKD transitioning from adolescence to early adulthood. Higher accuracy was observed with pediatric equations or those incorporating pediatric-specific variables such as age and height, whereas the adult equations tended to overestimate GFR and exhibited greater bias. Although most equations demonstrated strong correlations with mGFR, correlation reflects association rather than agreement and therefore cannot be interpreted as evidence of clinical accuracy or interchangeability. Accordingly, interpretation of equation performance in this study primarily relied on bias, precision, and accuracy metrics, including P10 and P30, which more directly reflect clinical reliability.

Among the equations evaluated, Schwartz_Cr_, U25, and FAS_Cr_-Ht demonstrated superior performance, showing lower bias and higher accuracy than the CKD-EPI equations. These findings are consistent with previous studies [6, 7, 24]. Notably, the U25_Cr−CysC_ and FAS_Cr_-Ht equations consistently exhibited low bias and high accuracy in both adolescents and young adults. Most of the better-performing equations include height as a key variable, highlighting its importance in GFR estimation during growth periods [1, 6, 8]. Height is a well-established surrogate marker of both kidney size and function in children and adolescents, and this physiological development does not abruptly cease at the age of 18 years [3, 6]. Many young adults remain in the transitional phase, with ongoing changes in body composition, muscle mass, and kidney physiology [13]. Abrupt switching between pediatric and adult equations at 18 years of age may disrupt the continuity and accuracy of kidney function assessment. The European Kidney Function Consortium reported a mean difference of 23 mL/min/1.72 m^2^ in eGFR values when switching from pediatric to adult equations during the transition to adult care [25]. Similarly, a longitudinal analysis in a separate study applying the Schwartz_Cr_ and CKD-EPI_Cr_ equations in patients aged 10 to 30 years showed poor agreement between the two equations during the transition period [5]. These findings support the use of equations designed to span childhood through adulthood in transitional care to ensure consistent monitoring of kidney function.

According to the KDIGO guidelines, an eGFR equation is considered clinically acceptable if it achieves 80–90% accuracy within 30% of the mGFR [11]. However, none of the equations evaluated in our study met this threshold. Importantly, the study population consisted entirely of Korean pediatric and young adult patients, whereas most eGFR equations were developed in Western cohorts. Although the U25 equations, developed from the CKiD cohort, have demonstrated 89–91% accuracy within 30% of mGFR across multiple racial groups in prior studies [8], they have not yet been externally validated in non-Western populations. In our study, 16.6% of person-visits were from patients with CKD stage 5, a subgroup in which all equations demonstrated poor performance, with an accuracy within 30%, ranging from 0% to 38.7%. These results are consistent with previous reports indicating reduced eGFR accuracy at mGFR levels < 30 mL/min/1.73 m^2^ [24, 26]. When CKD stage 5 visits were excluded, FAS_Cr_-Ht and Schwartz_Cr_ achieved accuracy within 30% rates of 84.6% and 84.0%, respectively. These findings underscore the need for externally validated GFR estimation equations tailored to pediatric and young adult populations, including those with advanced CKD, as emphasized in the 2024 KIDGO guidelines [11].

Cystatin C-based equations have undergone significant refinement since Schwartz introduced the improved eGFR equations for pediatric patients with CKD in 2012 [24, 27]. Unlike creatinine, which is affected by muscle mass, sex, and tubular reabsorption, cystatin C is produced at a constant rate, and is less influenced by non-GFR determinants [27]. It is particularly effective in reflecting GFR in pediatric CKD patients with moderate renal impairment (15–75 mL/min/1.73 m^2^) [3, 28]. The 2024 KDIGO guidelines recommend estimating GFR using a combination of creatinine and cystatin C, when available, as dual-marker equations offer greater accuracy than single-marker models [11]. In our study, the U25_Cr−CysC_ equation demonstrated the most balanced overall performance, and the CKD-EPI_Cr−CysC_ equation showed greater reliability than the CKD-EPI_Cr_ equation. Combined creatinine-cystatin C equations also exhibited tighter clustering of data points around the regression line and narrower limits of agreement, indicating superior consistency across the GFR spectrum. These findings provide supportive evidence that multi-biomarker eGFR equations may improve the accuracy and overall reliability of GFR estimation in patients with CKD transitioning from adolescence to early adulthood [3, 4, 23].

As observed in the Bland-Altman analysis, disagreement between eGFR and mGFR increased at higher mGFR values in this study. Such discrepancies are clinically relevant, particularly when important clinical decisions depend on accurate GFR estimation. Overestimation of eGFR in the higher GFR range may increase the risk of nephrotoxicity from medications or contrast agents, whereas underestimation may lead to unnecessary treatment restrictions or inappropriate exclusion during kidney donor evaluation. Consistent with these concerns, the 2024 KDIGO CKD guidelines recommend considering mGFR for critical decisions, such as kidney donor evaluation, drug dosing for medications with narrow therapeutic indices, and situations in which eGFR may be unreliable [11]. Given that our cohort primarily consisted of patients with moderate-to-severe CKD, data in the higher GFR range were limited; therefore, these findings should be interpreted with caution.

This study had several limitations. First, the relatively small sample size limited the statistical power, particularly for subgroup analyses. Second, the cohort included only Korean patients and predominantly consisted of adolescents and young adults with moderate to severe CKD, with a substantial proportion of visits occurring in CKD stages 3–5. As a result, the range of mGFR values was relatively narrow, which may have led to an overestimation of precision for several eGFR equations. Therefore, the precision and accuracy estimates observed in this study may not be generalizable to individuals with normal kidney function, hyperfiltration, or early-stage CKD. Third, mGFR was measured using two different tracers depending on availability at participating centers, which may have introduced tracer-related variability. Although tracer-specific standardized protocols were applied, external cross-center quality control was not performed, and residual inter-center variability cannot be entirely excluded. Fourth, serum creatinine was measured using a Jaffé rate blank method, although IDMS-traceable calibration was applied. The Jaffé method is susceptible to interference from non-creatinine chromogens, including proteins, glucose, and other serum constituents, which may result in positive bias, particularly at low creatinine concentrations [29]. This may have influenced the performance of creatinine-based and creatinine–cystatin C–based eGFR equations and could partially explain the observed differences in bias across CKD stages [30]. Enzymatic creatinine assays were not available in the central laboratory during the study period and therefore could not be evaluated as a comparator, although the use of IDMS-traceable calibration likely reduced systematic measurement error. Finally, despite the use of IFCC-certified reference material for cystatin C calibration in the central laboratory, minor residual calibration differences over time cannot be completely ruled out. The 2024 KDIGO guidelines strongly support the use of standardized, IFCC-traceable cystatin C assays to improve the reliability of eGFR measurements [11].

In this study of adolescents and young adults with CKD, eGFR equations covering children, particularly Schwartz_Cr_, U25_Cr−CysC_, and FAS_Cr_-Ht, demonstrated superior accuracy and lower bias than adult equations such as CKD-EPI. Equations incorporating pediatric-specific variables, particularly height, performed better during this transitional age period. Notably, none of the evaluated equations achieved the KDIGO-recommended accuracy threshold across the entire cohort, particularly in advanced CKD cases. These findings highlight the need for externally validated age-appropriate eGFR equations tailored to pediatric and young adult populations, including those with severe CKD, to ensure reliable assessment of kidney function during the transition from pediatric to adult care.

## Supplementary Information

Below is the link to the electronic supplementary material.

Supplementary Material 1

## Data Availability

Data are available from the corresponding author upon reasonable request.
