# Supplementary material for: Comparison of estimated and measured GFR in pediatric CKD patients transitioning from adolescence to adulthood: results from KNOW-PedCKD
Source: BMC Nephrol. 2026 Apr 2;27:306. doi: 10.1186/s12882-026-04942-w (PMC13169761; doi:10.1186/s12882-026-04942-w)

Supplementation 1. List of eGFR equations

In this study, U25_Cr_ equation, U25_cysC_ equation, U25_Cr-cysC_ equation, Schwartz_Cr_ equation, CKiD_Cr-CysC_ equation, FAS_Cr_-Age equation, FAS_Cr_-Ht equation, FAS_cysC_ equation, CKD-EPI_Cr_ equation and CKD-EPI_Cr-CysC_ equation were analyzed by comparing it with isotope GFR.

The U25 equation used in this study are as follows.

- U25_Cr_ age- and sex-dependent equation using S_cr_:

eGFR = κ × (height/S_cr_), with height in m and serum creatinine in mg/dL

For males, κ is calculated as:

For 1 to <12 years old: 39.0 × 1.008^(age-12)^

For 12 to <18 years old: 39.0 × 1.045^(age-12)^

For 18 to 25 years old: 50.8

For females, κ is calculated as:

For 1 to <12 years old: 36.1 × 1.008^(age-12)^

For 12 to <18 years old: 36.1 × 1.023^(age-12)^

For 18 to 25 years old: 41.4

- U25_CysC_ age- and sex-dependent equation using cysC:

eGFR = κ × (1/cysC), with serum cystatin C in mg/dL

For males, κ is calculated as:

For 1 to <15 years old: κ = 87.2 × 1.011^(age-15)^

For 15 to <18 years old: κ =87.2 × 0.960^(age-15)^

For 18 to 25 years old: κ = 77.1

For females, κ is calculated as:

For 1 to <12 years old: κ =79.9 × 1.004^(age-12)^

For 12 to <18 years old: κ = 79.9 × 0.974^(age-12)^

For 18 to 25 years old: κ = 68.3

- U25_Cr-CysC_ equation:

$eGFR=\frac{{U25}_{Cr}+{U25}_{CysC}}{2}$, mean based on serum creatinine level and serum cystatin C

The Schwartz equations used in this study are as follows.

- Schwartz_Cr_ equation (2009):

eGFR = 0.413 × (height/S_cr_), with height in cm and serum creatinine in mg/dL

- CKiD_Cr-CysC_ equation (2012):

eGFR = 39.8 × [ht/S_cr_]^0.456^× [1.8/cysC]^0.418^× [30/BUN]^0.079^× [1.076 male] [1.00 female] × [ht/1.4]^0.179^

Full Age spectrum (FAS) equation used in this study are as follows.

- FAS_Cr_-Age equation:

eGFR = 107.3/(S_cr_/Q), with serum creatinine in mg/dL

Q calculated as a function of age in years [Q(age)] as:

Males: Q = 0.21 + (0.057 × age) - (0.0075 × age^2^) + (0.00064 × age^3^) - (0.000016 × age^4^)

- FAS_Cr_-Ht equation:

eGFR = 107.3/(S_cr_/Q), with serum creatinine in mg/dL

Q calculated as a function of height in m [Q(height)] as:

All: Q = 3.94 - (13.4 × height) + (17.6 × height^2^) - (9.84 × height^3^) + (2.04 × height^4^)

- FAS_CysC_ equation using cysC:

eGFR = 107.3(cysC/Q), with serum cystatin C in mg/L and Q =0.82 for males and females < 70 years old

The CKD-EPI equations used in this study are as follows.

- CKD-EPI_Cr_ equation:

eGFR_cr_= 142 × min (S_cr_/κ, 1)^α^ × max(S_cr_/κ, 1)^-1.200^ × 0.9938^Age^ × [1.012 if female]

where S_cr_ is serum creatinine in mg/dL, κ is 0.7 for females and 0.9 for males, α is -0.241 for female and -0.302 for males, min is the minimum of S_cr_/ κ or 1, and max is the maximum of S_cr_/κ or 1.

- CKD-EPI_Cr-Cystatin C_ equation:

eGFR_cr-cys_= 135 × min (S_cr_/κ, 1)^α^× max (S_cr_/κ, 1)^-0.544^× min(S_cysC_/0.8, 1)^-0.323^× max(S_cysC_/0.8, 1)^-0.778^× 0.9961^Age^× 0.963 [if female]

where S_cr_ is serum creatinine, S_cysC_ is serum cystatin C, κ is 0.7 for females and 0.9 for males, α is -0.219 for females and -0.144 for males, min indicates the minimum of S_cr_/κ or 1, and max indicates the maximum of S_cr_/κ or 1.

Supplementation 2. Body surface area (BSA) formula used for mGFR indexing

The DuBois BSA formula used in this study is as follows:

- BSA (m^2^) = Weight (kg)^0.425^ × Height (cm)^0.725^ × 0.007184

Supplementary Table 1. ρ values of Spearman correlation for measured GFR vs. eGFR, stratified by sex, age and CKD stage

|  | Total | Sex | | Age | | CKD stage | | | | |
| --- | --- | --- | --- | --- | --- | --- | --- | --- | --- | --- |
|  |  | Male (n=142) | Female (n=45) | 15-18 yrs (n=83) | ≥ 18yrs (n=104) | Stage 1 (n=26) | Stage 2 (n=36) | Stage 3 (n=54) | Stage 4 (n=40) | Stage 5 (n=31) |
| U25_Cr_ | 0.96 | 0.95 | 0.98 | 0.96 | 0.96 | 0.51 | 0.56 | 0.68 | 0.50 | 0.64 |
| U25_CysC_ | 0.96 | 0.95 | 0.97 | 0.95 | 0.95 | 0.82 | 0.32^††^ | 0.67 | 0.60 | 0.72 |
| U25_Cr-CysC_ | 0.97 | 0.97 | 0.98 | 0.96 | 0.97 | 0.64 | 0.61 | 0.73 | 0.57 | 0.71 |
| Schwartz_Cr_ | 0.95 | 0.95 | 0.98 | 0.95 | 0.95 | 0.44^†^ | 0.48 | 0.65 | 0.50 | 0.56 |
| CKiD_Cr-CsyC_ | 0.97 | 0.96 | 0.99 | 0.96 | 0.97 | 0.65 | 0.51 | 0.77 | 0.57 | 0.66 |
| FAS_Cr_-Age | 0.93 | 0.91 | 0.98 | 0.95 | 0.91 | 0.53 | 0.55 | 0.60 | 0.50 | 0.64 |
| FAS_Cr_-Ht | 0.95 | 0.94 | 0.98 | 0.95 | 0.95 | 0.45^†^ | 0.36^†^ | 0.64 | 0.46 | 0.53 |
| FAS_CysC_ | 0.95 | 0.95 | 0.97 | 0.95 | 0.95 | 0.75 | 0.29^††^ | 0.67 | 0.60 | 0.57 |
| CKD-EPI_Cr_ | 0.96 | 0.94 | 0.98 | 0.96 | 0.95 | 0.41^†^ | 0.59 | 0.63 | 0.58 | 0.61 |
| CKD-EPI_Cr-CysC_ | 0.97 | 0.96 | 0.99 | 0.97 | 0.97 | 0.78 | 0.51 | 0.73 | 0.63 | 0.68 |

* ρ is Coefficient of Spearman correlation, ^†^*P* value < 0.05 ^††^*P* value > 0.05, otherwise *P* value < 0.01

Supplementary Table 2. Agreement and proportional bias of eGFR equations assessed using Bland-Altman analysis

|  | Limits of agreement (range) | Regression slope (95% CI) | R^2^ | *P* value |
| --- | --- | --- | --- | --- |
| U25_Cr_ | -18.86 to 23.99 (42.8) | -0.147 (-0.194 to -0.101) | 0.173 | <0.001 |
| U25_CysC_ | -23.87 to 26.23 (50.1) | -0.279 (-0.328 to -0.229) | 0.403 | <0.001 |
| U25_Cr-CysC_ | -18.70 to 22.44 (41.1) | -0.228 (-0.267 to -0.190) | 0.423 | <0.001 |
| Schwartz_Cr_ | -28.18 to 21.29 (49.5) | -0.233 (-0.284 to -0.181) | 0.300 | <0.001 |
| CKiD_Cr-CsyC_ | -17.47 to 24.10 (41.6) | -0.251 (-0.288 to -0.214) | 0.491 | <0.001 |
| FAS_Cr_-Age | -16.74 to 33.31 (50.1) | -0.021 (-0.078 to 0.036) | 0.003 | 0.465 |
| FAS_Cr_-Ht | -24.91 to 24.02 (48.9) | -0.162 (-0.217 to -0.108) | 0.158 | <0.001 |
| FAS_CysC_ | -15.28 to 34.78 (50.1) | -0.120 (-0.177 to -0.064) | 0.086 | <0.001 |
| CKD-EPI_Cr_ | -12.22 to 41.67 (53.9) | 0.148 (0.096 to 0.200) | 0.147 | <0.001 |
| CKD-EPI_Cr-CysC_ | -10.59 to 26.67 (37.3) | 0.060 (0.021 to 0.100) | 0.047 | 0.003 |

Limits of agreement were calculated as the mean difference (bias) ± 1.96 × standard deviation of the differences between eGFR and mGFR. Bias was defined as measured GFR minus eGFR. Regression slope represents proportional bias, derived from linear regression of the difference between eGFR and mGFR against the mean of measured GFR and eGFR. A regression slope significantly different from zero indicates the presence of proportional bias. R² represents the proportion of variance in the difference between eGFR and mGFR explained by the mean GFR. CI, confidence interval.

Supplementary Table 3. Regression analysis of absolute error for evaluation of heteroscedastic bias across eGFR equations

|  | Regression slope (95% CI) | R^2^ | *P* value |
| --- | --- | --- | --- |
| U25_Cr_ | 0.096 (0.066 to 0.126) | 0.173 | <0.001 |
| U25_CysC_ | 0.089 (0.049 to 0.129) | 0.095 | <0.001 |
| U25_Cr-CysC_ | 0.068 (0.037 to 0.098) | 0.093 | <0.001 |
| Schwartz_Cr_ | 0.184 (0.149 to 0.220) | 0.368 | <0.001 |
| CKiD_Cr-CsyC_ | 0.033 (0.003 to 0.063) | 0.025 | 0.032 |
| FAS_Cr_-Age | 0.076 (0.036 to 0.116) | 0.071 | <0.001 |
| FAS_Cr_-Ht | 0.143 (0.108 to 0.178) | 0.258 | <0.001 |
| FAS_CysC_ | -0.020 (-0.066 to 0.027) | 0.004 | 0.399 |
| CKD-EPI_Cr_ | 0.183 (0.141 to 0.227) | 0.275 | <0.001 |
| CKD-EPI_Cr-CysC_ | 0.094 (0.063 to 0.125) | 0.165 | <0.001 |

Absolute error was defined as the absolute difference between eGFR and mGFR. Regression slope represents the change in absolute error per unit increase in mean GFR. A positive slope indicates increasing variability in estimation error across higher levels of kidney function, consistent with heteroscedasticity. R² represents the proportion of variance in absolute error explained by the mean GFR. CI, confidence interval.

Supplementary Figure 1. Correlation between different eGFR and measured GFR.


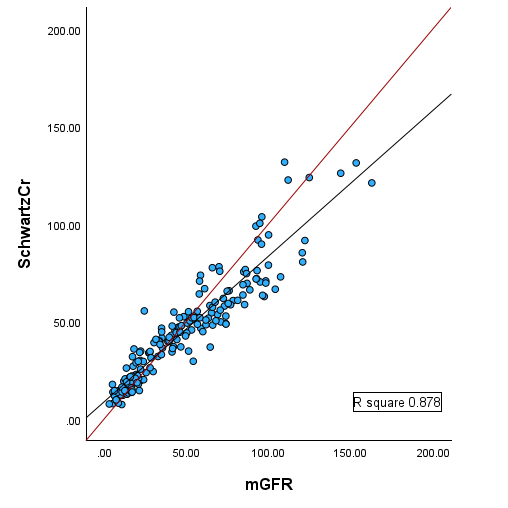

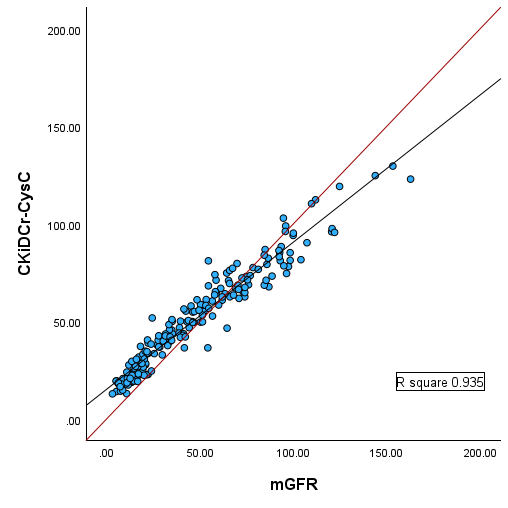

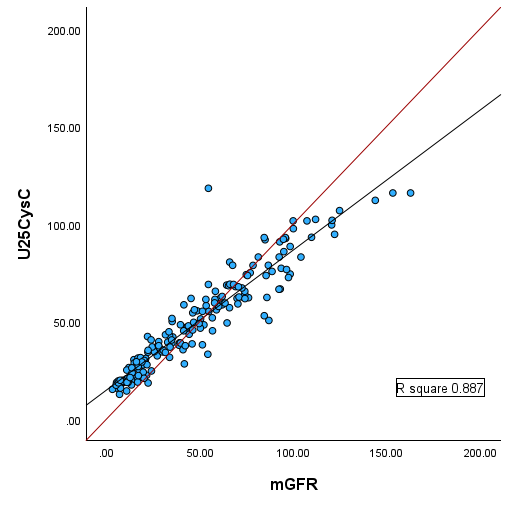

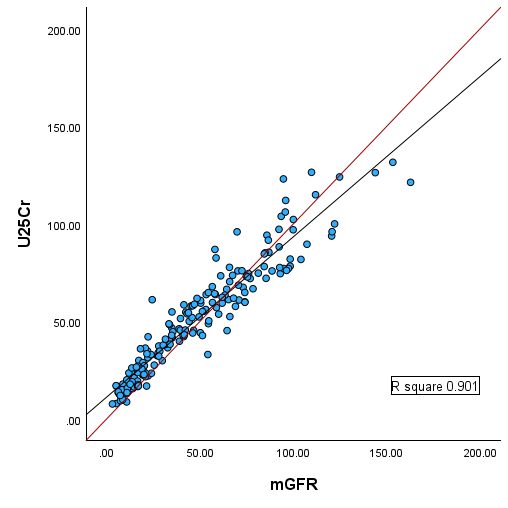

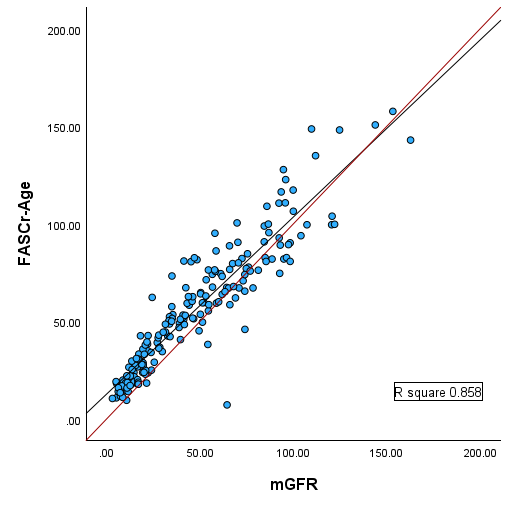

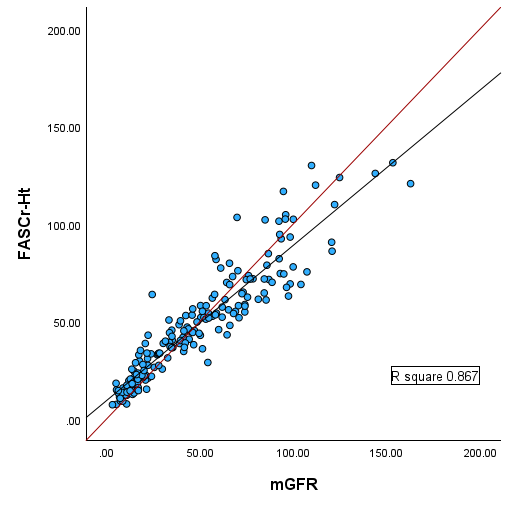

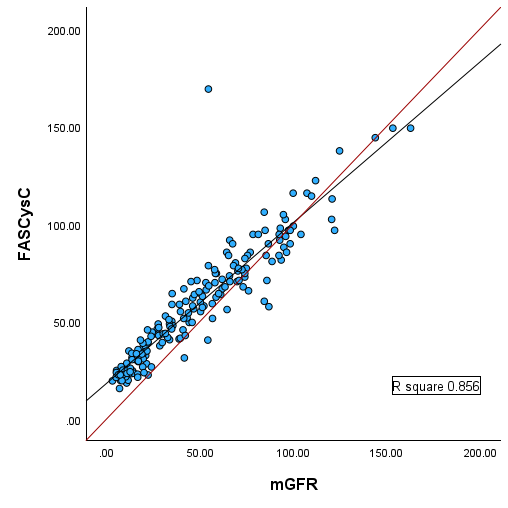

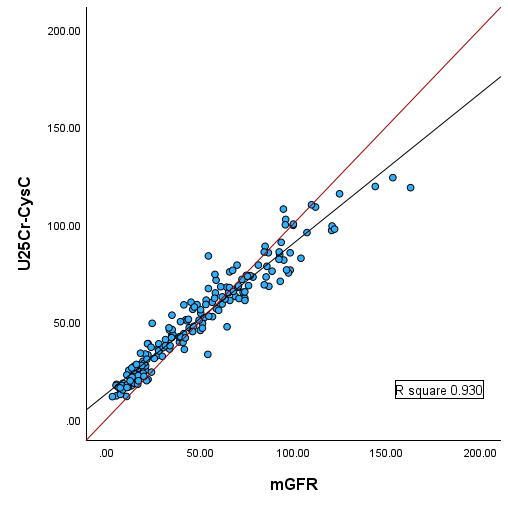

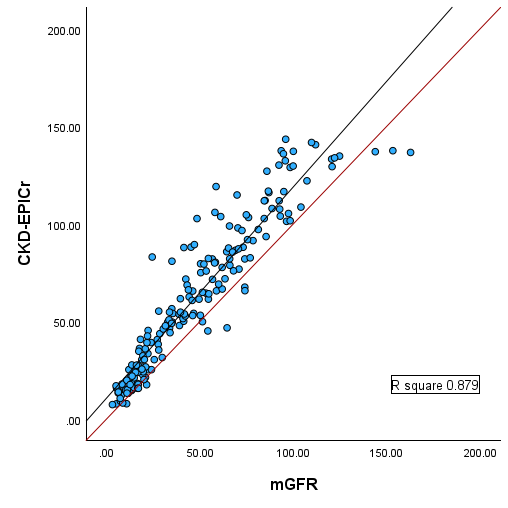

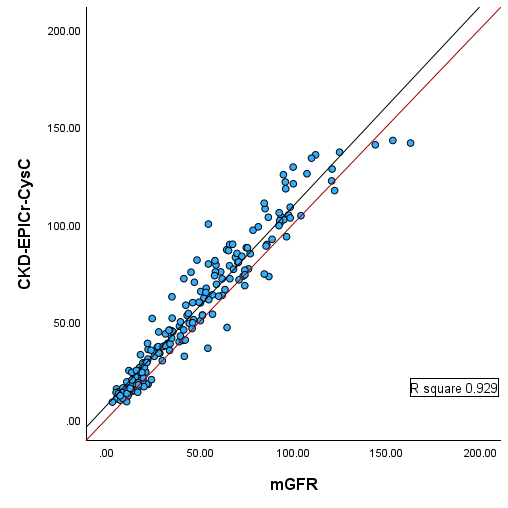

Supplement: Supplementary file 1 — Supplementary Material 1 [file 12882_2026_4942_MOESM1_ESM.docx]
